# Association between individual Warburg‐related proteins and prognosis in colorectal cancer

**DOI:** 10.1002/2056-4538.70016

**Published:** 2025-02-27

**Authors:** Kelly Offermans, Josien CA Jenniskens, Colinda CJM Simons, Iryna Samarska, Gregorio E Fazzi, Kim M Smits, Leo J Schouten, Matty P Weijenberg, Heike I Grabsch, Piet A van den Brandt

**Affiliations:** ^1^ Department of Epidemiology GROW – Research Institute for Oncology and Reproduction, Maastricht University Medical Center+ Maastricht The Netherlands; ^2^ Department of Pathology GROW – Research Institute for Oncology and Reproduction, Maastricht University Medical Center+ Maastricht The Netherlands; ^3^ Pathology and Data Analytics Leeds Institute of Medical Research at St James's, University of Leeds Leeds UK; ^4^ Department of Epidemiology Care and Public Health Research Institute (CAPHRI), Maastricht University Medical Center+ Maastricht The Netherlands

**Keywords:** Warburg effect, survival, prognosis, colorectal cancer, glycolysis

## Abstract

We previously showed that Warburg subtyping (low/moderate/high), based on the expression of six glycolytic proteins and transcriptional regulators [glucose transporter 1 (GLUT1), pyruvate kinase M2 (PKM2), lactate dehydrogenase A (LDHA), monocarboxylate transporter 4 (MCT4), p53, and PTEN], holds independent prognostic value in colorectal cancer (CRC) patients. The present study aimed to investigate whether the expression level of one of the proteins (GLUT1, PKM2, LDHA, MCT4, p53, and PTEN) can act as a proxy for our previously identified six protein‐based Warburg subtypes. Protein expression levels for individual Warburg‐related proteins were available for 2,251 CRC patients from the Netherlands Cohort Study. Kaplan–Meier curves and Cox regression were used to explore associations between individual Warburg‐related proteins and CRC‐specific and overall survival. Previously identified associations between Warburg subtypes and CRC‐specific and overall survival were adjusted for individual proteins, showing a significant association with survival in the current study. Multivariable‐adjusted analyses showed that the expression of GLUT1, LDHA, MCT4, PKM2, or p53 was associated with neither CRC‐specific nor overall survival. Decreasing PTEN expression was associated with significantly poorer overall survival (*p‐*trend_categories_ = 0.026). Additional adjustment for PTEN expression had minimal impact on the previously identified association between Warburg subtypes and survival, and the six protein‐based Warburg‐high subtype remained a statistically significant predictor of overall survival (hazard ratio 1.15; 95% CI 1.01–1.32). In conclusion, our results emphasise that individual Warburg‐related proteins cannot serve as a proxy or surrogate marker for Warburg subtyping, thereby highlighting the importance of combining the expression levels of multiple Warburg‐related proteins when examining the prognostic significance of a complex biological pathway such as the Warburg effect.

## Introduction

Despite advances in the early detection and treatment of colorectal cancer (CRC), it remains the world's second most deadly cancer accounting for more than 900,000 deaths in 2022 [1]. The tumour‐node‐metastasis (TNM) staging system remains the most commonly used clinical factor for predicting prognosis and guiding treatment decisions in CRC patients [2, 3, 4, 5]. However, patients with the same TNM stage can have different survival and/or response to adjuvant therapy, most likely due to heterogeneity in patient or tumour characteristics [4, 5, 6, 7, 8]. While numerous biomarkers have been investigated to improve prognostication in CRC patients [9], only a limited number have been translated to clinical practice [6, 9]. Hence, there remains an urgent clinical need to identify novel prognostic and/or predictive biomarker(s) to improve patient management decisions, leading to better survival of CRC patients [6, 9, 10].

Altered cellular metabolism has been identified as one of the hallmarks of cancer [11, 12]. The best‐known metabolic alteration is the ‘Warburg effect’, a phenomenon characterised by increased glucose uptake and lactate excretion in the presence of oxygen (i.e. aerobic glycolysis) [13]. Previous research has shown that the phosphoinositide 3‐kinase (PI3K) / protein kinase B (AKT) / mammalian target of rapamycin (mTOR) pathway induces the Warburg effect by stimulating several key processes [14, 15, 16, 17, 18, 19]. These include upregulation of glucose transporter 1 (GLUT1) expression to enhance glucose uptake [18, 20], increased expression of pyruvate kinase M2 (PKM2) and lactate dehydrogenase A (LDHA) to boost glycolytic flux and lactate production [17, 18], and upregulation of monocarboxylate transporter 4 (MCT4) expression to facilitate lactate excretion [18, 20]. It has been suggested that this so‐called ‘glycolytic switch’ is driven by the loss of critical transcription factors, such as p53 or PTEN [21, 22].

A relationship between the expression level of individual proteins related to the Warburg effect and CRC patients' survival has been suggested in the past, but results have been inconsistent [23]. In a previous study, we showed that Warburg subtyping (i.e. Warburg‐low, Warburg‐moderate, and Warburg‐high), based on the combined expression level of four glycolytic proteins (GLUT1, PKM2, LDHA, and MCT4) and two transcriptional regulators (p53 and PTEN), had prognostic value independent of established prognostic factors like TNM stage [24].

Immunohistochemical staining for six markers is both expensive and time‐consuming, making it impractical for high‐throughput large‐scale studies and unlikely to be implemented in routine clinical practice. Hence, the overall aim of the present study was to investigate whether the expression level of any individual Warburg‐related protein (GLUT1, PKM2, LDHA, MCT4, p53, and PTEN) can act as a proxy for Warburg subtyping. We therefore (1) investigated the associations between individual protein expression levels, CRC‐specific and overall survival, and (2) determined whether any of the individual Warburg‐related proteins can serve as a proxy for Warburg subtyping in a large prospective series of CRC patients (*n* = 2,251).

## Materials and methods

### Study design and population

The population‐based series of CRC patients analysed in this study was derived from the Netherlands Cohort Study (NLCS) on diet and cancer, which is a prospective study that has previously been described in detail [25]. The NLCS was initiated in September 1986 and included 120,852 men and women, aged 55–69 at baseline [25]. Participants completed a mailed, self‐administered questionnaire at baseline on diet and other risk factors for cancer [25].

**Figure 1:**
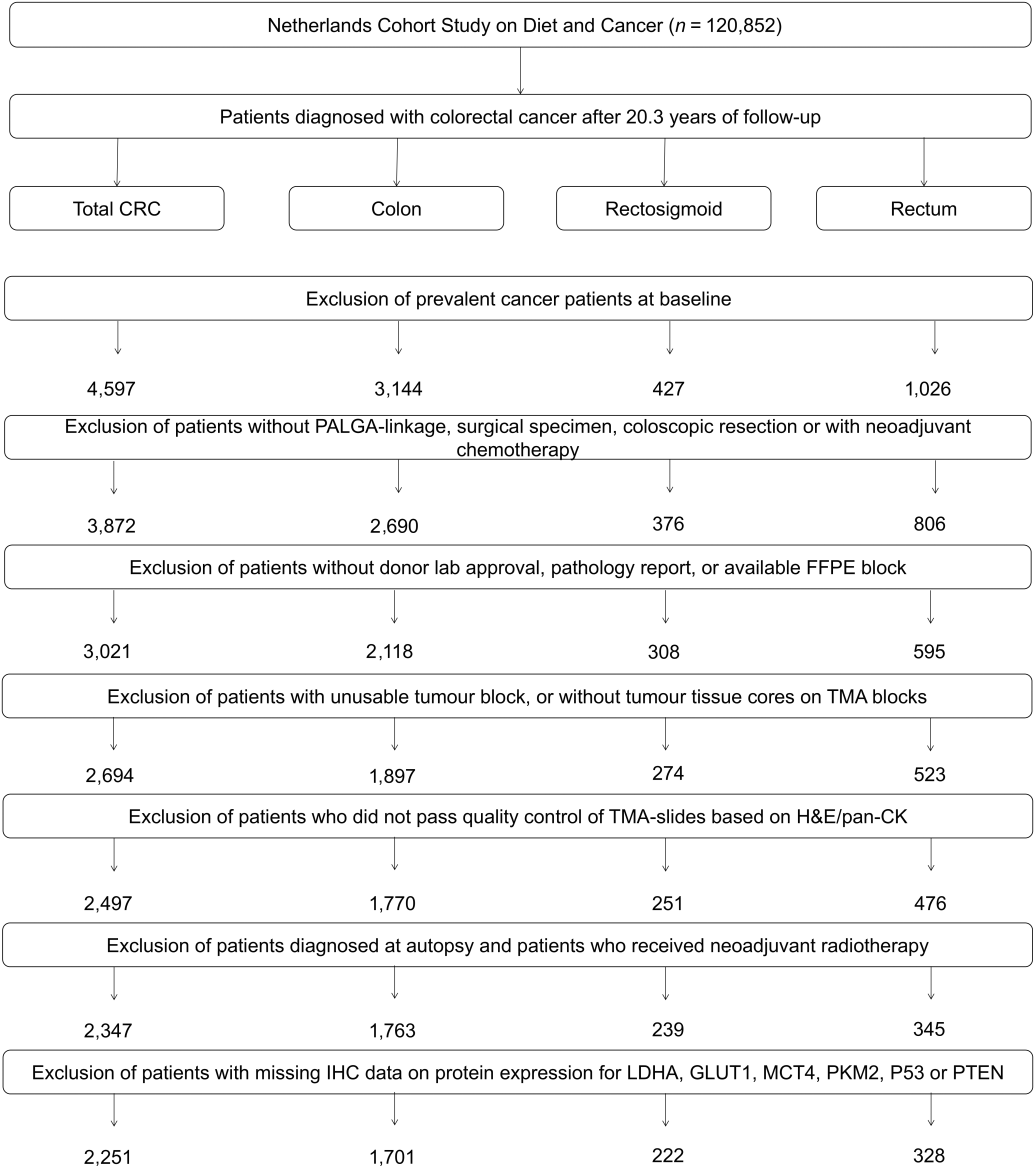
Flow diagram of the number of incident colorectal cancer patients available for analyses in the Netherlands Cohort Study (NLCS), 1986–2006.

All participants were followed up for cancer incidence by annual record linkage with the Netherlands Cancer Registry and the Dutch Pathology Registry (PALGA) [26], covering 20.3 years of follow‐up (17 September 1986 until 1 January 2007). It was estimated that the completeness of cancer incidence follow‐up was >96% [27]. Patients who reported a history of cancer (excluding non‐melanoma skin cancer) at baseline were excluded from analyses, resulting in a total of 4,597 incident CRC patients available for analyses (Figure 1).

The NLCS was approved by the institutional review boards of the TNO Quality of Life Research Institute (Zeist, the Netherlands) and Maastricht University (Maastricht, the Netherlands). All cohort members consented to participation by completing the questionnaire.

### Tissue collection and tissue microarray construction

During 2012–2017, formalin‐fixed paraffin‐embedded (FFPE) blocks from incident CRC patients within the NLCS were collected as part of the Rainbow Tissue MicroArray (TMA) project [28]. In total, 78 TMA blocks were constructed, each containing three 0.6 mm tissue cores with tumour and three cores with matched normal mucosa from 2,694 CRC patients (for details see [24]). Ethical approval was granted by the Medical Ethical Committee (METC) of Maastricht University Medical Center+, Maastricht, the Netherlands.

### Immunohistochemistry

Prior to this study, TMA blocks were cut to obtain serial tissue sections (5 μm). These sections were subjected to haematoxylin and eosin (H&E) staining or immunohistochemistry (IHC) for six glycolytic proteins (LDHA, GLUT1, MCT4, PKM2, p53, and PTEN) and mismatch‐repair (MMR) proteins (MLH1 and MSH2), as described in detail previously [24]. A total of 2,497 CRC patients with at least one tumour core per patient passed quality control (Figure 1).

Patients diagnosed at autopsy (*n* = 5) and patients who received neoadjuvant radiotherapy (*n* = 145) were excluded, leaving 2,347 CRC patients for Warburg subtyping (see Figure 1). The IHC scoring protocols and the process of combining multiple core‐level scores into patient‐level scores were previously described in detail (see supplementary figure S1 and figure 2 in Jenniskens *et al* [29]). In short, patient‐level protein expression data were derived by averaging scores from one to three tumour cores per patient and rounding to the nearest scoring category [24]. Based on these patient‐level scores, patients were categorised with low, moderate, or high protein expression for all six individual Warburg‐related proteins (LDHA, GLUT1, MCT4, PKM2, p53, and PTEN) [24]. For PTEN and p53, cut‐offs were derived from published literature [30, 31], while cut‐offs for other proteins were based on the patient distribution (see supplementary table S4 in Jenniskens *et al* [29] for cut‐offs for individual proteins) [24]. The expression levels of these six proteins were combined into a pathway‐based sum score, where high expression of p53, GLUT1, LDHA, MCT4, or PKM2 contributed 2 points per protein, moderate expression 1 point, and low expression 0 points [24]. PTEN, inversely associated with the Warburg effect, had its scoring reversed (2 = low expression; 0 = high expression). The sum score ranged from 0 to 12, with higher scores indicating a stronger involvement of the Warburg effect [24]. Patients with missing data for any of the Warburg‐related proteins were excluded, leaving 2,251 CRC patients classified into Warburg‐low (score 0–3, *n* = 652), Warburg‐moderate (score 4–5, *n* = 802), and Warburg‐high (score 6–12, *n* = 797) subtypes (Figure 1).

MMR status was evaluated following the protocol of Richman *et al* [30]. Tumours with loss of either MLH1 or MSH2 expression, in the presence of internal positive controls, were identified as MMR deficient (dMMR), while tumours that expressed both MLH1 and MSH2 were considered MMR proficient (pMMR).

### Clinical characteristics and follow‐up

Data on patient and tumour characteristics, including age at diagnosis, pathological tumour‐node‐metastasis (pTNM) stage, tumour location, differentiation grade, and initial treatment information, were obtained from the cancer registry or PALGA histopathology reports, as appropriate. Follow‐up for vital status was established through linkage with the Central Bureau of Gynaecology and the municipal population registries until 31 December 2012. Causes of death were obtained from Statistics Netherlands. Vital status was available for 2,250 patients (>99.9%), and information regarding CRC‐specific death was available for 2,216 patients (98.5%).

### Statistical analyses

Descriptive statistics and frequency distributions were calculated for Warburg subtypes and individual Warburg‐related proteins. Differences between individual Warburg‐related proteins were evaluated using chi‐square tests (categorical variables) or Kruskal–Wallis tests (continuous variables). Primary endpoints of interest included CRC‐specific survival (time from CRC diagnosis to CRC‐related death or end of follow‐up) and overall survival (time from CRC diagnosis to death from any cause or end of follow‐up). Survival analyses were restricted to 10 years of follow‐up, because of the limited number of events in the later period (CRC‐specific deaths: *n* = 32; overall deaths: *n =* 268).

Univariable and multivariable‐adjusted Cox proportional hazards regression were used to (1) investigate associations with survival according to protein expression levels (low/moderate/high) of the individual Warburg‐related protein(s) (i.e. GLUT1, LDHA, MCT4, PKM2, p53, PTEN), and (2) to investigate whether any individual Warburg‐related protein could serve as a surrogate marker for Warburg subtyping.

The proportional hazards assumption was tested using the scaled Schoenfeld residuals [32], by evaluating log‐log‐transformed survival curves and by introducing time–covariate interactions into the models. All multivariable models were adjusted for a set of *a priori* selected prognostic factors, as described previously [24]: age at diagnosis (years), sex (men and women), tumour location (colon, rectosigmoid, and rectum), pTNM stage (I, II, III, and IV), differentiation grade (well, moderate, and poor/undifferentiated), adjuvant therapy (no and yes), and dMMR (no and yes). A separate category (‘unknown’) was used for patients with unknown clinical information regarding pTNM stage, differentiation grade, adjuvant therapy or dMMR, to enable inclusion of these patients in the Cox proportional hazards regression models. To address the second aim, multivariable models to investigate associations between Warburg subtypes and survival were additionally adjusted for any individual Warburg‐related protein(s) that showed significant associations with survival. Potential additional confounders were year of diagnosis (per 3 years) and used TNM version. These potential confounders were retained in the final models only if they introduced a ≥10% change in hazard ratios (HRs) [33, 34].

Disease stage was based on the pTNM classification according to the edition valid at the time of cancer diagnosis, resulting in the use of five different TNM editions Union for International Cancer Control (UICC) TNM editions 3–6), as described previously [24]. However, the main TNM stage groupings (I/II/III/IV) have remained essentially unchanged [35]. In a second analysis, multivariable models were additionally adjusted for expression levels of all other individual Warburg‐related proteins.

All analyses were conducted in Stata Statistical Software: Release 16 (StataCorp, College Station, TX, USA). The *p* values <0.05 were considered statistically significant.

## Results

### Correlations between individual Warburg‐related proteins and Warburg subtypes

Spearman's rank correlation coefficients between the individual Warburg‐related proteins are shown in supplementary material, Table S1. Negligible correlations were observed between expression levels of individual Warburg‐related proteins, except for a moderate correlation between LDHA and MCT4 (Spearman's rank correlation coefficient: +0.33). Spearman's rank correlation coefficients between individual Warburg‐related proteins and Warburg subtypes ranged from +0.18 for PTEN expression to +0.51 for PKM2 expression.

### Clinical characteristics

Clinical characteristics of the incident CRC patients (*n* = 2,251) from the NLCS according to protein expression level (low, moderate, and high) of the individual glycolytic proteins (LDHA, GLUT1, MCT4, and PKM2) and transcriptional regulators (p53 and PTEN) are presented in Tables 1 and 2, respectively.

**Table 1:** Clinical characteristics of the total series of colorectal cancer patients within the Netherlands Cohort Study (NLCS; 1986–2006), as well as according to the expression levels (low, moderate, and high) of glycolytic proteins associated with the Warburg effect (LDHA, GLUT1, MCT4, and PKM2)

		GLUT1 (*n* = 2,251)				LDHA (*n* = 2,251)				MCT4 (*n* = 2,251)				PKM2 (*n* = 2,251)			
		Low	Moderate	High	*p* *	Low	Moderate	High	*p* *	Low	Moderate	High	*p* *	Low	Moderate	High	*p* *
Clinical characteristics	Total (*n* = 2,251)	(*n* = 945)	(*n* = 778)	(*n* = 528)		(*n* = 781)	(*n* = 1,075)	(*n* = 395)		(*n* = 1,145)	(*n* = 633)	(*n* = 473)		(*n* = 1,049)	(*n* = 598)	(*n* = 604)	
Age at diagnosis in years, median (range)	74.0 (55.0–89.0)	74.0 (55.0–89.0)	74.0 (56.0–88.0)	74.0 (56.0–89.0)	0.499^†^	73.0 (55.0–89.0)	74.0 (56.0–89.0)	75.0 (56.0–88.0)	**0.003** ^†^	74.0 (55.0–89.0)	74.0 (56.0–89.0)	74.0 (57.0–88.0)	**0.043** ^†^	74.0 (55.0–89.0)	74.0 (56.0–88.0)	74.0 (56.0–89.0)	0.549^†^
Sex, %																	
Men	55.2	53.8	57.7	54.0	0.212	54.6	58.5	47.3	**0.001**	58.2	55.6	47.4	**<0.001**	59.2	55.0	48.3	**<0.001**
Women	44.8	46.2	42.3	46.0		45.5	41.5	52.7		41.8	44.4	52.6		40.8	45.0	51.7	
Tumour location, %																	
Colon	75.6	76.5	74.6	75.4	0.874	74.5	73.4	83.5	**0.002**	72.8	73.6	84.8	**<0.001**	72.5	76.1	80.5	**0.009**
Rectosigmoid	9.9	9.2	10.3	10.4		10.8	10.5	6.3		11.0	10.3	6.6		11.3	9.5	7.8	
Rectum	14.6	14.3	15.2	14.2		14.7	16.1	10.1		16.2	16.1	8.7		16.3	14.4	11.8	
pTNM stage, %																	
I	18.8	22.5	18.9	12.1	**<0.001**	18.7	20.2	15.4	0.397	18.3	20.2	18.4	0.070	20.0	19.9	15.7	0.188
II	38.6	38.1	40.1	37.1		38.8	37.5	41.0		36.8	38.4	43.1		37.8	38.0	40.6	
III	25.9	24.1	24.3	31.3		26.6	25.0	26.6		26.8	27.0	22.0		26.1	25.3	26.0	
IV	14.4	12.6	14.3	18.0		13.2	15.0	15.4		15.8	12.2	14.2		12.8	16.1	15.7	
Unknown	2.3	2.7	2.4	1.5		2.7	2.3	1.5		2.4	2.2	2.3		3.3	0.8	2.0	
Tumour extension (pT), %																	
T1	4.2	5.8	3.6	2.3	**<0.001**	3.8	5.1	2.5	0.063	3.8	4.4	5.1	0.522	4.4	4.7	3.5	0.442
T2	18.0	19.8	19.3	13.1		18.1	19.1	15.2		18.1	19.8	15.6		18.4	19.6	15.9	
T3	64.7	62.0	64.1	70.5		65.9	63.2	66.6		64.9	63.5	66.0		64.0	64.2	66.6	
T4	10.4	9.4	10.2	12.3		9.5	10.0	13.2		10.8	9.3	10.6		9.5	10.2	11.9	
Unknown	2.7	3.0	2.8	1.9		2.7	2.7	2.5		2.5	3.0	2.8		3.7	1.3	2.2	
Lymph node involvement (pN), %																	
N0	52.2	53.9	54.5	45.6	**<0.001**	52.4	52.3	51.4	0.793	50.0	53.9	55.2	0.143	52.0	53.0	51.7	0.593
N+	36.3	33.4	34.1	44.9		35.7	36.1	38.2		37.9	35.4	33.8		34.9	36.5	38.7	
Unknown	11.5	12.7	11.4	9.5		11.9	11.6	10.4		12.1	10.7	11.0		13.2	10.5	9.6	
Differentiation grade, %																	
Well	8.9	9.7	8.7	7.8	**<0.001**	10.5	8.5	7.1	0.048	10.7	7.6	6.6	**<0.001**	11.2	8.0	6.0	**<0.001**
Moderate	65.7	64.9	69.8	61.0		66.5	66.4	62.0		67.1	66.7	60.9		66.7	65.9	63.6	
Poor/undifferentiated	17.6	15.9	15.3	24.2		15.9	17.5	21.5		15.0	18.0	23.5		13.4	18.6	24.2	
Unknown	7.8	9.5	6.2	7.0		7.2	7.6	9.4		7.3	7.7	9.1		8.8	7.5	6.3	
dMMR, %																	
No	87.7	83.7	91.0	90.2	**<0.001**	92.1	89.7	73.9	**<0.001**	94.7	88.5	70.0	**<0.001**	91.5	87.0	82.0	**<0.001**
Yes	11.2	15.1	8.4	8.5		6.2	9.8	25.3		4.5	10.7	28.3		7.6	12.2	16.6	
Unknown	1.0	1.2	0.6	1.3		1.8	0.6	0.8		0.9	0.8	1.7		0.9	0.8	1.5	
Adjuvant therapy, %																	
No	83.3	84.6	83.9	79.9	**0.015**	83.5	82.8	84.1	0.742	82.3	82.9	86.1	0.183	82.8	83.1	84.3	0.912
Yes	15.8	14.1	15.2	19.9		15.2	16.5	15.2		16.7	16.3	13.1		15.8	16.2	15.4	
Unknown	0.9	1.4	0.9	0.2		1.3	0.7	0.8		1.1	0.8	0.9		1.4	0.7	0.3	

*Note*: Statistically significant *p* values are shown in bold font.

* The *p* value for the chi‐square test, unless otherwise specified.

^†^ The *p* value for the Kruskal–Wallis test

**Table 2:** Clinical characteristics of the total series of colorectal cancer patients within the Netherlands Cohort Study (NLCS; 1986–2006), as well as according to the expression levels (low, moderate, and high) of transcriptional regulators associated with the Warburg effect (p53 and PTEN)

		p53 (*n* = 2,251)				PTEN (*n* = 2,251)			
		Low	Moderate	High	*p* *	Low	Moderate	High	*p* *
Clinical characteristics	Total (*n* = 2,251)	(*n* = 1,114)	(*n* = 252)	(*n* = 885)		(*n* = 187)	(*n* = 1,171)	(*n* = 893)	
Age at diagnosis in years, median (range)	74.0 (55.0–89.0)	74.0 (56.0–89.0)	74.0 (55.0–88.0)	74.0 (55.0–89.0)	0.799^†^	74.0 (57.0–87.0)	73.0 (55.0–89.0)	75.0 (56.0–89.0)	**<0.001** ^†^
Sex, %									
Men	55.2	53.3	50.8	58.8	**0.017**	47.1	56.5	55.2	0.056
Women	44.8	46.7	49.2	41.2		52.9	43.6	44.8	
Tumour location, %									
Colon	75.6	77.5	82.9	71.1	**<0.001**	85.0	74.9	74.5	**0.030**
Rectosigmoid	9.9	9.8	5.2	11.3		6.4	10.5	9.7	
Rectum	14.6	12.8	11.9	17.6		8.6	14.6	15.8	
pTNM stage, %									
I	18.8	19.8	14.3	18.9	0.183	12.3	16.3	23.5	**<0.001**
II	38.6	39.7	42.1	36.2		41.7	37.9	38.8	
III	25.9	25.0	23.8	27.5		26.2	27.3	23.9	
IV	14.4	13.6	16.7	14.8		17.1	15.8	12.1	
Unknown	2.3	1.8	3.2	2.7		2.7	2.7	1.8	
Tumour extension (pT), %									
T1	4.2	3.8	4.8	4.6	0.197	0.5	2.8	6.8	**<0.001**
T2	18.0	19.1	12.7	18.2		14.4	16.8	20.4	
T3	64.7	64.4	66.3	64.8		73.3	65.1	62.5	
T4	10.4	10.8	12.7	9.2		8.6	12.5	8.0	
Unknown	2.7	2.0	3.6	3.3		3.2	2.8	2.4	
Lymph node involvement (pN), %									
N0	52.2	53.2	55.6	49.8	0.190	46.0	50.9	55.1	**0.007**
N+	36.3	35.0	34.9	38.4		40.6	38.8	32.3	
Unknown	11.5	11.8	9.5	11.8		13.4	10.3	12.7	
Differentiation grade, %									
Well	8.9	9.5	6.8	8.8	0.190	7.0	9.2	9.0	0.677
Moderate	65.7	63.5	67.1	68.0		64.2	67.4	63.7	
Poor/undifferentiated	17.6	19.1	16.7	16.1		20.9	17.4	17.3	
Unknown	7.8	7.9	9.5	7.1		8.0	6.0	10.1	
dMMR, %									
No	87.7	82.7	79.4	96.5	**<0.001**	80.2	92.7	82.8	**<0.001**
Yes	11.2	15.7	20.2	3.1		17.7	5.8	17.0	
Unknown	1.0	1.6	0.4	0.5		2.1	1.5	0.2	
Adjuvant therapy, %									
No	83.3	84.7	80.2	82.3	0.132	86.1	81.6	84.8	0.105
Yes	15.8	14.4	18.7	16.8		12.3	17.3	14.7	
Unknown	0.9	0.9	1.2	0.9		1.6	1.1	0.6	

*Note*: Statistically significant *p* values are shown in bold font.

* The *p* value for the chi‐square test, unless otherwise specified.

^†^ The *p* value for the Kruskal–Wallis test.

#### Glycolytic proteins

Patients with GLUT1_high_ CRC were more frequently diagnosed with an advanced disease stage (pTNM stage III–IV), increased depth of tumour invasion in the wall (pT3–T4), lymph node metastases (pN+), poorly or undifferentiated tumours, and were more frequently treated with adjuvant therapy in comparison to patients with GLUT1_low_ CRC (Table 1). Moreover, patients with GLUT1_low_ CRC more often had dMMR compared to patients with GLUT1_high_ CRC (Table 1).

Patients with LDHA_high_ or MCT4_high_ CRC were older at time of diagnosis compared to patients with LDHA_low_ and MCT4_low_ CRC (Table 1). Patients with LDHA_high_, MCT4_high_, or PKM2_high_ CRC more frequently had tumours located in the colon, poorly or undifferentiated tumours, and dMMR compared to patients with LDHA_low_, MCT4_low_, or PKM2_low_ CRC (Table 1), respectively.

For detailed information with regard to clinical characteristics of patients with GLUT1_moderate_, LDHA_moderate_, MCT4_moderate_, or PKM2_moderate_ CRC, refer to Table 1.

#### Transcriptional regulators

Patients with p53_high_ CRC were more frequently male compared to patients with p53_low_ CRC (Table 2). Furthermore, patients with p53_high_ CRC more often had tumours located in the rectum or rectosigmoid, and more often showed pMMR compared to patients with p53_low_ CRC (Table 2).

Patients with PTEN_low_ CRC were younger at the time of diagnosis in comparison to patients with PTEN_high_ CRC (Table 2). In addition, patients with PTEN_low_ CRC more often had tumours located in the colon and were more frequently diagnosed with advanced disease stage (pTNM III–IV), increased depth of tumour invasion in the wall (pT3–T4), and lymph node metastasis (pN+) compared to patients with PTEN_high_ CRC (Table 2). Lastly, patients with either PTEN_low_ or PTEN_high_ CRC were more likely to have dMMR than patients with PTEN_moderate_ CRC (Table 2).

Detailed information with regard to clinical characteristics of patients with p53_moderate_ or PTEN_moderate_ CRC are presented in Table 2.

### Survival

The median follow‐up time since diagnosis was 4.79 years (range 0.0027–25.99 years). Survival analyses were restricted to 10 years of follow‐up. During these first 10 years of follow‐up, 1,463 deaths were observed, of which 933 (63.8%) were CRC‐related deaths.

#### Univariable analyses

Univariable Kaplan–Meier curves showed significant differences in overall and/or CRC‐specific survival of patients according to protein expression levels of GLUT1, MCT4, PKM2, and PTEN (supplementary material, Figures S1 and S2, respectively). In univariable analyses (Table 3), increasing GLUT1 expression was associated with a statistically worse overall survival (*p‐*trend_categories_ < 0.001) and CRC‐specific survival (*p‐*trend_categories_ < 0.001). Furthermore, patients with decreasing PTEN expression showed significantly worse overall (*p‐*trend_categories_ = 0.001) and CRC‐specific survival (*p‐*trend_categories_ < 0.001). Patients with increasing PKM2 expression showed worse CRC‐specific survival (*p‐*trend_categories_ = 0.038). Lastly, patients with increasing MCT4 expression showed significantly better CRC‐specific survival (*p‐*trend_categories_ = 0.038).

**Table 3:** Univariable and multivariable‐adjusted hazard ratios (HRs) and 95% confidence intervals (CIs) for associations between the expression levels (low, moderate, and high) of individual Warburg‐related proteins (LDHA, GLUT1, MCT4, PKM2, p53, and PTEN), and CRC‐specific and overall survival

		CRC‐specific survival				Overall survival			
			HR (95% CI)			Deaths (%)	HR (95% CI)		
	*N*	CRC deaths (%)	Univariable	Multivariable‐adjusted*	Multivariable‐adjusted^†^		Univariable	Multivariable‐adjusted*	Multivariable‐adjusted^†^
Warburg subtypes									
Low	652	245 (37.6)	1.00 (ref)	1.00 (ref)	1.00 (ref)^‡^	402 (61.7)	1.00 (ref)	1.00 (ref)	1.00 (ref)^‡^
Moderate	802	339 (42.3)	1.16 (0.98–1.36)	1.04 (0.88–1.22)	1.03 (0.87–1.21)^‡^	514 (64.1)	1.07 (0.94–1.22)	1.01 (0.89–1.15)	1.00 (0.88–1.14)^‡^
High	797	349 (43.8)	1.27 (1.08–1.50)	1.17 (0.99–1.38)	1.14 (0.96–1.35)^‡^	547 (68.6)	1.24 (1.08–1.41)	1.18 (1.03–1.34)	1.15 (1.01–1.32)^‡^
*p*‐trend			0.004	0.061	0.116^‡^		0.001	0.012	0.030^‡^
GLUT1									
Low	945	346 (36.6)	1.00 (ref)	1.00 (ref)	1.00 (ref)	590 (62.4)	1.00 (ref)	1.00 (ref)	1.00 (ref)
Moderate	778	332 (42.7)	1.22 (1.05–1.42)	1.16 (1.00–1.35)	1.13 (0.97–1.32)	503 (64.7)	1.09 (0.97–1.23)	1.07 (0.95–1.21)	1.05 (0.93–1.18)
High	528	255 (48.3)	1.46 (1.24–1.71)	1.17 (0.99–1.38)	1.15 (0.97–1.36)	370 (70.1)	1.27 (1.11–1.44)	1.09 (0.96–1.25)	1.07 (0.93–1.23)
*p*‐trend			<0.001	0.047	0.089		<0.001	0.162	0.303
LDHA									
Low	781	331 (42.4)	1.00 (ref)	1.00 (ref)	1.00 (ref)	500 (64.0)	1.00 (ref)	1.00 (ref)	1.00 (ref)
Moderate	1,075	452 (42.0)	1.00 (0.87–1.15)	0.99 (0.85–1.14)	0.99 (0.85–1.15)	706 (65.7)	1.04 (0.93–1.17)	1.02 (0.91–1.14)	1.02 (0.91–1.15)
High	395	150 (38.0)	0.89 (0.73–1.08)	0.91 (0.75–1.11)	0.92 (0.74–1.13)	257 (65.1)	1.01 (0.87–1.18)	1.01 (0.86–1.17)	1.02 (0.87–1.20)
*p*‐trend			0.302	0.388	0.471		0.745	0.876	0.762
MCT4									
Low	1,145	509 (44.5)	1.00 (ref)	1.00 (ref)	1.00 (ref)	762 (66.6)	1.00 (ref)	1.00 (ref)	1.00 (ref)
Moderate	633	241 (38.1)	0.82 (0.71–0.96)	0.95 (0.81–1.10)	0.96 (0.81–1.12)	404 (63.8)	0.92 (0.82–1.04)	1.01 (0.89–1.14)	1.01 (0.89–1.15)
High	473	183 (38.7)	0.85 (0.72–1.00)	1.00 (0.84–1.20)	1.04 (0.86–1.26)	297 (62.8)	0.91 (0.80–1.04)	1.01 (0.88–1.16)	1.02 (0.88–1.19)
*p*‐trend			0.018	0.868	0.848		0.116	0.862	0.799
PKM2									
Low	1,049	410 (39.1)	1.00 (ref)	1.00 (ref)	1.00 (ref)	666 (63.5)	1.00 (ref)	1.00 (ref)	1.00 (ref)
Moderate	598	263 (44.0)	1.18 (1.01–1.38)	1.14 (0.97–1.33)	1.13 (0.96–1.32)	393 (65.7)	1.10 (0.97–1.24)	1.06 (0.94–1.21)	1.06 (0.94–1.21)
High	604	260 (43.0)	1.16 (0.99–1.36)	1.12 (0.95–1.31)	1.08 (0.92–1.28)	404 (66.9)	1.12 (0.99–1.26)	1.10 (0.97–1.25)	1.07 (0.94–1.22)
*p‐*trend			0.038	0.137	0.284		0.062	0.135	0.255
p53									
Low	1,114	446 (40.0)	1.00 (ref)	1.00 (ref)	1.00 (ref)	711 (63.8)	1.00 (ref)	1.00 (ref)	1.00 (ref)
Moderate	252	112 (44.4)	1.13 (0.92–1.39)	1.18 (0.96–1.46)	1.17 (0.95–1.45)	161 (63.9)	1.02 (0.86–1.21)	1.07 (0.90–1.27)	1.06 (0.89–1.26)
High	885	375 (42.4)	1.08 (0.94–1.24)	0.98 (0.85–1.12)	0.96 (0.84–1.11)	591 (66.8)	1.07 (0.96–1.19)	1.00 (0.89–1.12)	0.99 (0.89–1.11)
*p*‐trend			0.268	0.772	0.642		0.226	0.990	0.919
PTEN									
Low	187	81 (43.3)	1.36 (1.06–1.73)	1.20 (0.94–1.54)	1.16 (0.90–1.49)	123 (65.8)	1.22 (1.01–1.49)	1.18 (0.97–1.44)	1.18 (0.96–1.44)
Moderate	1,171	531 (45.3)	1.36 (1.18–1.56)	1.14 (0.99–1.31)	1.13 (0.97–1.31)	797 (68.1)	1.22 (1.10–1.36)	1.13 (1.01–1.26)	1.13 (1.01–1.27)
High	893	321 (35.9)	1.00 (ref)	1.00 (ref)	1.00 (ref)	543 (60.8)	1.00 (ref)	1.00 (ref)	1.00 (ref)
*p*‐trend			<0.001	0.053	0.108		0.001	0.022	0.026

* Adjusted for *a priori* defined confounders, including age at diagnosis (years), sex (male and female), tumour location (colon, rectum, and rectosigmoid), pTNM stage (I, II, III, IV, and unknown), differentiation grade (well, moderate, poor/undifferentiated, and unknown), MMR deficiency (no, yes, and unknown), adjuvant therapy (no, yes, and unknown).

^†^ Additionally adjusted for the expression levels of all individual proteins (LDHA: low, moderate, and high; GLUT1: low, moderate, and high; PKM2: low, moderate, and high; MCT4: low, moderate, and high; PTEN: low, moderate, and high; p53: low, moderate, and high), unless otherwise specified.

^‡^ Associations between Warburg subtypes (low, moderate, and high) and CRC‐specific and overall survival were adjusted for *a priori* defined confounders, including age at diagnosis (years), sex (male and female), tumour location (colon, rectum, and rectosigmoid), pTNM stage (I, II, III, IV, and unknown), differentiation grade (well, moderate, poor/undifferentiated, and unknown), MMR deficiency (no, yes, and unknown), adjuvant therapy (no, yes, and unknown), and additionally adjusted for PTEN expression (low, moderate, and high).

#### Multivariable‐adjusted analyses

After adjusting for *a priori* defined confounders (age at diagnosis, sex, tumour location, pTNM stage, differentiation grade, MMR status, and adjuvant therapy status), only GLUT1 and PTEN remained significant predictors of CRC‐specific or overall survival, respectively (Table 3). Patients with increasing GLUT1 expression showed significantly poorer CRC‐specific survival (*p‐*trend_categories_ = 0.047). Furthermore, patients with decreasing PTEN expression showed a significantly worse overall survival (*p*‐trend_categories_ = 0.022). Additional adjustment for the expression levels of all individual Warburg‐related proteins (Table 3) showed that only decreasing PTEN expression remained a significant predictor of overall survival (*p‐*trend_categories_ = 0.026).

To explore whether PTEN expression could serve as a surrogate marker for Warburg subtyping, Cox regression models examining the association between Warburg subtypes and CRC‐specific and overall survival were additionally adjusted for PTEN expression (Table 3). Additional adjustment for PTEN expression did not significantly affect the association between the Warburg‐high subtype and either CRC‐specific (HR_PTEN‐unadjusted_ 1.17; 95% CI 0.99–1.38 versus HR_PTEN‐adjusted_ 1.14; 95% CI 0.96–1.35; Table 3) or overall survival (HR_PTEN‐unadjusted_ 1.18; 95% CI 1.03–1.34 versus HR_PTEN‐adjusted_ 1.15; 95% CI 1.01–1.32; Table 3), and the Warburg‐high subtype remained a significant predictor of overall survival.

#### Stratified analyses: tumour location and pTNM stage

As previously published [24], the Warburg‐high subtype was associated with a significantly worse survival in patients with tumours located in the rectum or pTNM stage III CRC. Stratified analyses showed that none of the individual Warburg‐related proteins were significantly associated with CRC‐specific or overall survival in patients with cancers located in the rectum (supplementary material, Tables S2 and S3). Furthermore, none of the individual Warburg‐related proteins were associated with CRC‐specific survival in patients with pTNM stage III CRC (supplementary material, Table S4). Of all individual Warburg‐related proteins studied, only decreasing PTEN expression was significantly associated with worse overall survival in pTNM stage III CRC (*p*‐trend_categories_ = 0.008; supplementary material, Table S5).

## Discussion

We have recently introduced Warburg subtyping (Warburg‐low, Warburg‐moderate, and Warburg‐high), based on the expression levels of six glycolytic proteins and transcriptional regulators indicative of the presence of the Warburg effect (GLUT1, PKM2, LDHA, MCT4, p53, and PTEN), as a promising novel prognostic [24] and predictive [36] marker in CRC patients. Since immunohistochemical staining for six markers is rather costly and labour‐intensive, it is not feasible for large‐scale, high‐throughput studies and is unlikely to be implemented in routine clinical practice. Therefore, the current study aimed to investigate whether the expression of any individual Warburg‐related protein can act as a proxy for Warburg subtyping. Our results show for the first time that none of the individual Warburg‐related proteins can serve as a proxy or surrogate marker for Warburg subtyping, thereby highlighting the importance of combining the expression levels of multiple Warburg‐related proteins when examining the prognostic significance of a complex biological pathway such as the Warburg effect. More specifically, multivariable‐adjusted analyses revealed that high expression of GLUT1, LDHA, MCT4, PKM2, or p53 individually was not significantly associated with CRC‐specific or overall survival. Additional adjustment for expression levels of all individual proteins did not substantially change associations with survival, which was expected based on the negligible correlations we observed among individual proteins. Although we did observe a significant association between decreasing PTEN expression and poor overall survival, no significant association with CRC‐specific survival was observed (possibly due to limited power). Furthermore, decreasing PTEN expression alone could not explain the observed associations with poor survival for the Warburg‐high subtype in cancers located in the rectum or in pTNM stage III CRC [24]. To further explore whether PTEN expression could serve as a surrogate marker for Warburg subtyping, Cox regression models examining the association between Warburg subtypes and CRC‐specific and overall survival were additionally adjusted for PTEN expression. Additional adjustment did not significantly affect the association with survival we observed for the Warburg subtypes, and the Warburg‐high subtype remained a statistically significant predictor of overall survival.

### Glycolytic proteins related to the Warburg effect

GLUT1 plays a crucial role in facilitating glucose uptake and fuelling glycolysis in cancer cells [37]. Overexpression of GLUT1 has been identified in various cancer types and was shown to contribute to enhanced aerobic glycolysis [38]. Resulting from its important role in oncogenesis, several studies were conducted to investigate the prognostic value of GLUT1 in tumours [38], including CRC [39]. In line with our results, a previously conducted meta‐analysis, which included eight observational studies, reported no significant association between GLUT1 expression and overall and disease‐free survival in CRC [39].

LDHA catalyses the conversion of pyruvate to lactate and is considered a key checkpoint of glycolysis [40]. Enhanced LDHA expression has been related to tumour aggressiveness (i.e. promoting angiogenesis, immune evasion, extracellular matrix degradation, and tumour cell migration) as well as metastatic disease [41], and has therefore been proposed as a potential promising prognostic biomarker for cancers [42]. To the best of our knowledge, only one previous study has examined the prognostic value of IHC expression of LDHA in CRC [43]. Contrary to our results, the authors found that patients with high LDHA expression had a significantly poorer overall survival compared with those having low LDHA expression [43]. This difference may be explained by the fact that their study only included patients with TNM stage II and III CRC, whereas our study included patients with TNM stage I–IV CRC. Furthermore, it has been reported previously that high LDH serum levels were associated with poor overall survival (see [44] for meta‐analyses), though this meta‐analysis did not specifically investigate the LDHA isoform.

MCT4 is a lactate exporter that is overexpressed in highly glycolytic tissues [45, 46, 47]. High MCT4 expression is associated with tumour growth and infiltration [48], and is therefore thought to have potential prognostic value [49]. Several studies have investigated this in CRC, though the number of patients included in most of the studies was relatively low (range: *n* = 58–107) [50, 51, 52]. In line with our results, two previous studies found no statistically significant association between high tumoral MCT4 expression and overall survival in CRC [50, 53]. In contrast, two other studies found that high MCT4 expression was associated with poorer overall survival in CRC [51, 52]. The discrepancy in results may be explained by the fact that these previous studies stained for MCT4 using a less specific polyclonal antibody (H‐90, sc‐50329) which may have resulted in cross‐reaction with other proteins, whereas we used a highly specific monoclonal antibody (D‐1, sc‐376140).

Pyruvate kinase (PK) is a rate‐limiting glycolytic enzyme that catalyses the final step in glycolysis [54]. The PK muscle isozyme M2 (PKM2) has been shown to be increased in the majority of cancer cells [54]. Furthermore, PKM2 has been associated with tumour development and metastasis [55]. To the best of our knowledge, only one previous study has investigated the prognostic value of PKM2 in CRC [56]. In contrast to our results, the authors reported that high PKM2 expression was associated with a significantly poorer overall survival [56]. The discrepancy in results may again be caused by the different types of antibodies used. However, despite our efforts, we were unable to identify the specific characteristics of the antibody employed by the authors based on the information that was provided in the manuscript.

### Transcriptional regulators of the Warburg effect

In addition to the elevated expression of the above‐mentioned glycolytic proteins, various key transcriptional regulators such as p53 and PTEN have been shown to play important roles in the Warburg effect [57].

Tumour suppressor p53 is frequently mutated in various types of cancer [58], including CRC [59]. Wild‐type p53 is well known to prevent tumour development by controlling proliferation, motility, and survival of abnormal or stressed cells [60, 61]. More recently, it has been suggested that loss of p53 function contributes to the Warburg effect [61]. In line with our results, a previous meta‐analysis showed no prognostic value for *TP53* mutation in CRC [62]. More recently, another prospective study found that increased IHC p53 expression was associated with poorer overall survival in CRC [63]. However, different cut‐offs for p53 positivity were used compared to the present study, which might explain the discrepancy in reported results (positive: >55% versus negative: ≤55%, respectively). Furthermore, in line with our results the authors found no significant association between *TP53* mutational status and overall survival [63]. Nevertheless, a more robust investigation of p53 activity is required to understand whether p53 expression and activity may have prognostic value in CRC.

Lastly, phosphatase and tensin homologue (PTEN) inactivation has been identified in the vast majority of cancer types, including CRC [64]. PTEN has an essential role in regulating the PI3K/AKT signalling pathway, and is known to regulate apoptosis, cell growth, cell migration, cell cycle regulation, and tumour progression [64]. In addition, loss of PTEN has been associated with the Warburg effect [57]. The role of PTEN in CRC prognosis remains controversial. In line with our results, a previous study showed that loss of PTEN expression was associated with poor overall survival in CRC [65]. In contrast, other studies reported no significant association between loss of PTEN expression and overall survival [64, 66], though one of these studies did find a non‐significant positive association between loss of PTEN expression and overall survival [64].

### Individual proteins versus Warburg subtypes

In our previous study, we demonstrated that Warburg subtyping, based on the expression of Warburg‐related proteins (LDHA, GLUT1, MCT4, PKM2, p53, and PTEN), holds prognostic value in CRC, independent of well‐known prognostic factors such as TNM stage [24]. This was particularly notable for patients with pTNM stage III CRC and cancers located in the rectum [24]. Although the relationship we observed between Warburg subtyping and overall survival may be partly explained by loss of PTEN expression, associations between the Warburg‐high subtype and worse CRC‐specific survival or between the Warburg‐high subtype and poorer survival in rectal cancer patients cannot be solely attributed to PTEN loss as these associations were not observed for PTEN expression. To investigate the potential use of PTEN expression as a surrogate for Warburg subtyping, we examined the association between Warburg subtypes and survival after adjusting for PTEN expression. Additional adjustment for PTEN expression did not have substantial impact on the association between the Warburg subtypes and survival that we initially observed. Notably, the Warburg‐high subtype continued to be a statistically significant predictor of overall survival even after adjusting for PTEN expression, suggesting that PTEN expression alone cannot serve as a surrogate for Warburg subtyping. All in all, these results indicate that the sum of a number of variables (i.e. Warburg subtyping) may be more than the sum of its parts (i.e. individual Warburg‐related proteins).

Furthermore, these results suggest that PTEN may affect the survival of CRC patients through a biological mechanism other than the Warburg effect. Indeed, it has been described that, in addition to regulating the Warburg effect, PTEN is a multifunctional protein exerting a wide range of biological activities, including cell migration, cell adhesion to surrounding tissues, new blood vessel formation, and genetic stability [67].

### Current use of biomarker panels in CRC and future possibilities

The TNM staging system remains the primary tool used to predict prognosis and guide treatment decisions in CRC [4, 5, 68], with additional factors such as vascular, lymphatic, or nerve invasion, poor differentiation, advanced tumour stage (pT4), and inadequate lymph node sampling, aiding risk assessment in early‐stage CRC [68, 69]. Molecular markers (e.g. *KRAS, NRAS*, *BRAF* mutations, and MMR defects) have improved treatment selection, particularly for metastatic CRC, but their use in non‐metastatic CRC is limited [68]. RNA expression‐based approaches, such as Complexity INdex in SARComas (CINSARC) [70] or consensus molecular subtypes (CMS) [71], show promise but face challenges regarding clinical implementation, such as high costs, reliance on bioinformatics, and limited accuracy on FFPE tissue samples [72]. Hence, there is a need for cost‐effective, reliable biomarkers that ideally also serve as potential therapeutic targets. Our recent work demonstrates that Warburg subtyping can be relatively easily performed on FFPE tissue sections using IHC [24, 73], a laboratory technique that is already routinely used in clinical practice. Future research should focus on validating our results and/or further exploration of the added prognostic and predictive value of Warburg subtyping and individual Warburg‐related proteins. Furthermore, it would be of interest to explore the subcellular localisation of these individual Warburg‐related proteins when assessing their prognostic value, as it has been reported previously that this may affect observed outcomes [74].

### Strengths and limitations

This study possesses notable strengths, including its large population‐based series of CRC patients, the nearly complete follow‐up, and the fact that the large majority of patients were treated with surgery only. However, several potential limitations should be acknowledged. First, the selection of six Warburg‐related proteins in our study represents a subset of proteins involved in the pathway. Nevertheless, we believe that the chosen proteins offer a reasonably comprehensive understanding of the Warburg effect, as they encompass various levels of the pathway, including transcriptional regulation (PTEN, p53), glucose transport (GLUT1), glycolysis (PKM2, LDHA), and lactate secretion (MCT4). A second potential limitation concerns the utilisation of TMAs, which may not capture the entirety of the tumour. However, previous studies have demonstrated the reliability of triplicate 0.6 mm cores as a viable alternative for high‐throughput molecular profiling using IHC, when compared to whole‐tissue sections [75]. Lastly, limitations with regard to Warburg subtyping have been described previously [24].

## Conclusion

Our results show for the first time that none of the individual Warburg‐related proteins can serve as a proxy for Warburg subtyping, thereby highlighting the importance of combining the expression levels of multiple Warburg‐related proteins when examining the prognostic significance of a complex biological pathway such as the Warburg effect. Future research is necessary to validate our results and to further examine the prognostic value of Warburg subtyping over that of individual Warburg‐related proteins.

## Author contributions statement

PAvdB, HIG, CCJMS, JCAJ and KO conceived the study. PAvdB carried out methodology. PAvdB and LJS acquired data. KO carried out formal analysis and investigation. PAvdB, HIG, JCAJ and KO prepared the original manuscript draft. PAvdB, HIG, CCJMS, MPW, LJS, KMS, IS, GEF and JCAJ reviewed and edited the manuscript. PAvdB and HIG acquired funding and supervised the study. The work reported in the paper has been performed by the authors, unless clearly specified in the text.

## Supporting information


**Figure S1.** Univariable Kaplan–Meier curves showing CRC‐specific survival of colorectal cancer patients within the Netherlands Cohort Study (NLCS, 1986–2006) according to protein expression levels of GLUT1, LDHA, MCT4, PKM2, p53 and PTEN
**Figure S2.** Univariable Kaplan–Meier curves showing overall survival of colorectal cancer patients within the Netherlands Cohort Study (NLCS, 1986–2006) according to protein expression levels of GLUT1, LDHA, MCT4, PKM2, p53 and PTEN
**Table S1.** Spearman correlations between the individual Warburg‐related proteins (LDHA, GLUT1, MCT4, PKM2, p53, and reversed PTEN) as well as with Warburg subtypes
**Table S2.** Univariable and multivariable‐adjusted hazard ratios and 95% confidence intervals for associations between the expression levels of individual Warburg‐related proteins (LDHA, GLUT1, MCT4, PKM2, p53, and PTEN) and CRC‐specific survival, according to tumour location
**Table S3.** Univariable and multivariable‐adjusted hazard ratios and 95% confidence intervals for associations between the expression levels of individual Warburg‐related proteins (LDHA, GLUT1, MCT4, PKM2, p53, and PTEN) and overall survival, according to tumour location
**Table S4.** Univariable and multivariable‐adjusted hazard ratios and 95% confidence intervals for associations between the expression levels of individual Warburg‐related proteins (LDHA, GLUT1, MCT4, PKM2, p53, and PTEN) and CRC‐specific survival, according to disease stage
**Table S5.** Univariable and multivariable‐adjusted hazard ratios and 95% confidence intervals for associations between the expression levels of individual Warburg‐related proteins (LDHA, GLUT1, MCT4, PKM2, p53, and PTEN) and overall survival, according to disease stage

## Data Availability

The datasets generated and/or analysed during the current study are not publicly available because the informed consent does not allow for that.
