# Supplementary material for: Association between individual Warburg‐related proteins and prognosis in colorectal cancer
Source: J Pathol Clin Res. 2025 Feb 27;11(2):e70016. doi: 10.1002/2056-4538.70016 (PMC11868443; doi:10.1002/2056-4538.70016)
Supplement: Supplementary file 1 — Figure S1. Univariable Kaplan–Meier curves showing CRC‐specific survival of colorectal cancer patients within the Netherlands Cohort Study (NLCS, 1986–2006) according to protein expression levels of GLUT1, LDHA, MCT4, PKM2, p53 and PTEN Figure S2. Univariable Kaplan–Meier curves showing overall survival of colorectal cancer patients within the Netherlands Cohort Study (NLCS, 1986–2006) according to protein expression levels of GLUT1, LDHA, MCT4, PKM2, p53 and PTEN Table S1. Spearman correlations between the individual Warburg‐related proteins (LDHA, GLUT1, MCT4, PKM2, p53, and reversed PTEN) as well as with Warburg subtypes Table S2. Univariable and multivariable‐adjusted hazard ratios and 95% confidence intervals for associations between the expression levels of individual Warburg‐related proteins (LDHA, GLUT1, MCT4, PKM2, p53, and PTEN) and CRC‐specific survival, according to tumour location Table S3. Univariable and multivariable‐adjusted hazard ratios and 95% confidence intervals for associations between the expression levels of individual Warburg‐related proteins (LDHA, GLUT1, MCT4, PKM2, p53, and PTEN) and overall survival, according to tumour location Table S4. Univariable and multivariable‐adjusted hazard ratios and 95% confidence intervals for associations between the expression levels of individual Warburg‐related proteins (LDHA, GLUT1, MCT4, PKM2, p53, and PTEN) and CRC‐specific survival, according to disease stage Table S5. Univariable and multivariable‐adjusted hazard ratios and 95% confidence intervals for associations between the expression levels of individual Warburg‐related proteins (LDHA, GLUT1, MCT4, PKM2, p53, and PTEN) and overall survival, according to disease stage [file CJP2-11-e70016-s001.pdf]

Association between individual Warburg-related proteins and prognosis in colorectal cancer

K Offermans *et al*. *J Pathol Clin Res* https://doi.org/10.1002/2056-4538.70016

Supplementary Figures S1–S2

Supplementary Tables S1–S5

The reference number refers to the list in the main paper.

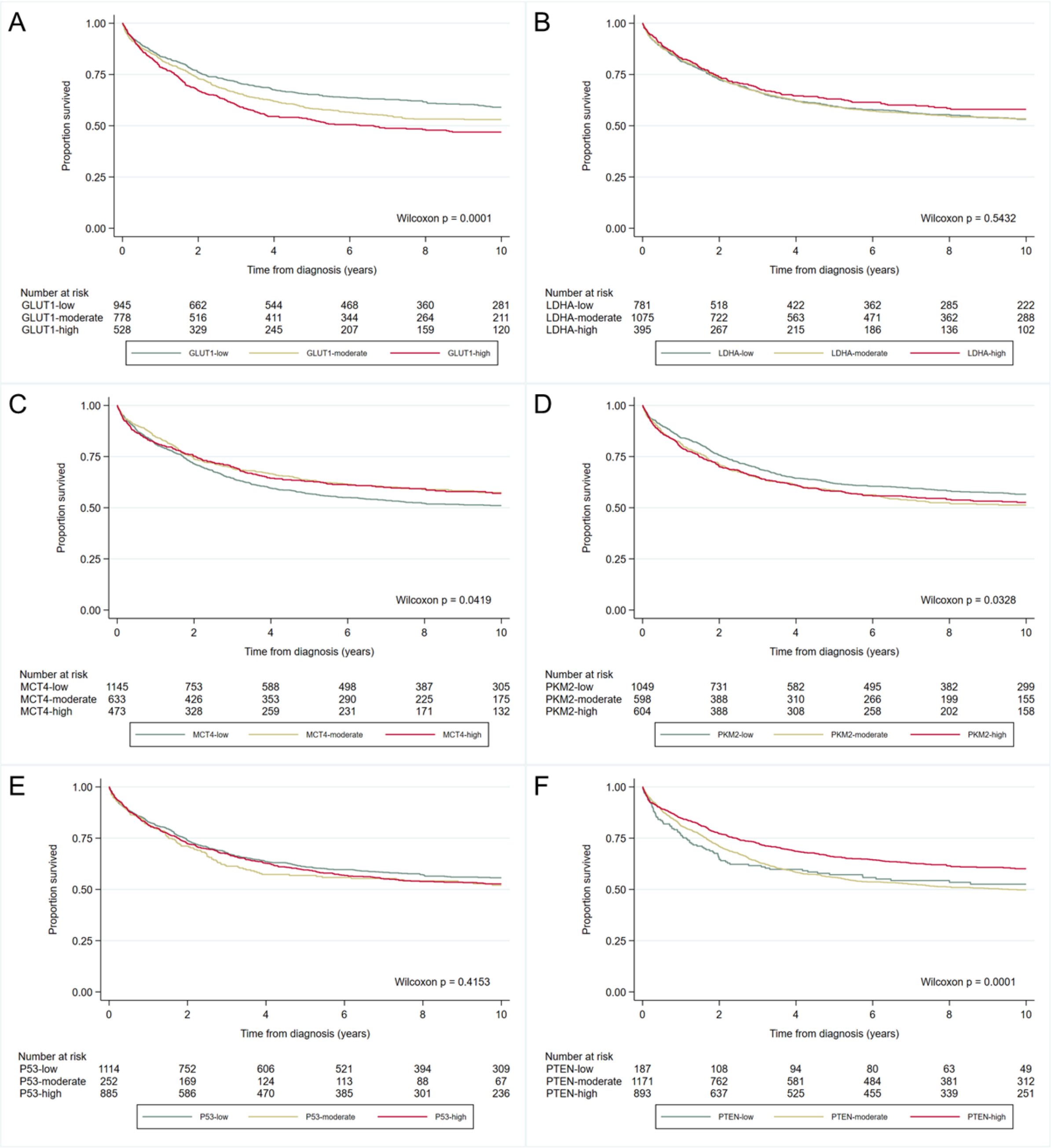

Figure S1. Univariable Kaplan–Meier curves showing CRC-specific survival of colorectal cancer patients within the Netherlands Cohort Study (NLCS, 1986–2006) according to protein expression levels (low, moderate, and high) of (A) GLUT1, (B) LDHA, (C) MCT4, (D) PKM2, (E) p53, and (F) PTEN.

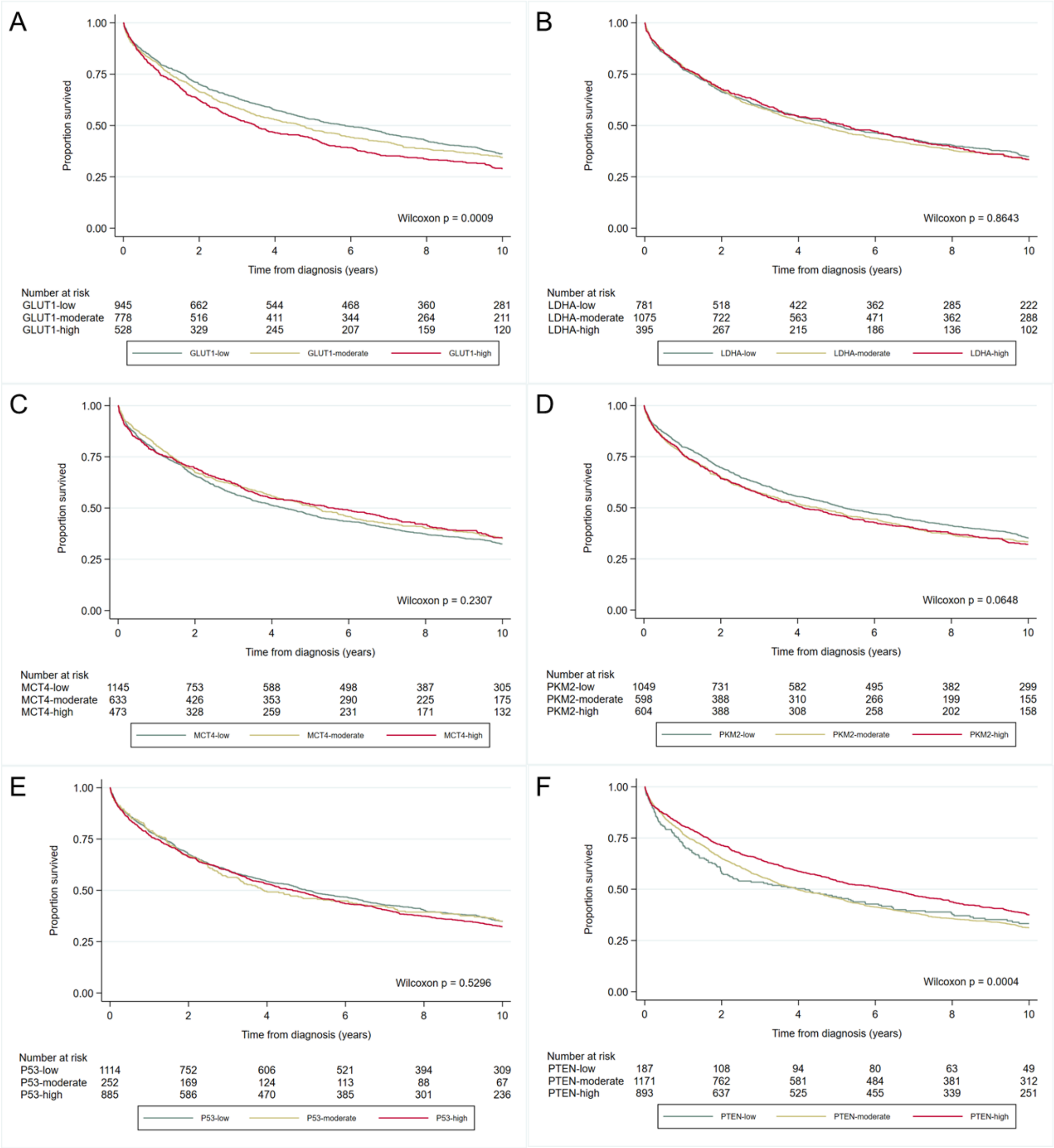

Figure S2. Univariable Kaplan–Meier curves showing overall survival of colorectal cancer patients within the Netherlands Cohort Study (NLCS, 1986-2006) according to protein expression levels (low, moderate, high) of (A) GLUT1, (B) LDHA, (C) MCT4, (D) PKM2, (E) p53, and (F) PTEN.

Table S1. Spearman correlations between the individual Warburg-related proteins (LDHA, GLUT1, MCT4, PKM2, p53, and reversed PTEN) as well as with Warburg subtypes.

	GLUT1	LDHA	MCT4	PKM2	p53	PTEN (rev)	Warburg subtypes
**GLUT1**	1.00						
**LDHA**	-0.03	1.00					
**MCT4**	0.01	0.33	1.00				
**PKM2**	0.15	0.12	0.17	1.00			
**p53**	0.08	0.01	-0.05	-0.03	1.00		
**PTEN (rev)**	0.10	-0.17	-0.19	0.01	0.03	1.00	
**Warburg-subtypes**	0.45	0.41	0.45	0.51	0.41	0.18	1.00

Interpretation of correlation coefficients was as follows: 0.01–0.19, no or negligible relationship; 0.20–0.29, weak relationship; 0.30–0.39, moderate relationship; 0.40–0.69, strong relationship; ≥0.70, very strong relationship; as described by Leclezio *et al* [76]. If the correlation coefficient is a positive number, the expression levels of the proteins are directly related (i.e. if the expression value of one protein goes up, the expression value of the other also tends to do so); if the coefficient is a negative number, the expression levels of the proteins are inversely related (i.e. as the expression value of one proteins goes up, the expression value of the other protein tends to go down).

Table S2. Univariable and multivariable-adjusted hazard ratios (HRs) and 95% confidence intervals (CIs) for associations between the expression levels (low, moderate, and high) of individual Warburg-related proteins (LDHA, GLUT1, MCT4, PKM2, p53, and PTEN) and CRC-specific survival, according to tumour location (colon, rectosigmoid, and rectum).

		Colon				Rectosigmoid				Rectum			
				HR (95% CI)				HR (95% CI)				HR (95% CI)	
		N	CRC deaths (%)	Univariable	Multivariable-adjusted[1]	N	CRC deaths (%)	Univariable	Multivariable-adjusted[1]	N	CRC deaths (%)	Univariable	Multivariable-adjusted[1]
**Warburg subtypes**													
	Low	467	183 (39.2)	1.00 (ref)	1.00 (ref)[2]	77	21 (27.3)	1.00 (ref)	1.00 (ref)[2]	108	41 (38.0)	1.00 (ref)	1.00 (ref)[2]
	Moderate	606	263 (43.4)	1.15 (0.95-1.38)	1.05 (0.87-1.27)	77	25 (32.5)	1.21 (0.68-2.16)	1.01 (0.55-1.83)	119	51 (42.9)	1.10 (0.73-1.67)	0.89 (0.58-1.36)
	High	628	268 (42.7)	1.14 (0.95-1.38)	1.14 (0.94-1.38)	68	36 (52.9)	2.69 (1.57-4.62)	1.69 (0.93-3.06)	101	45 (44.6)	1.38 (0.90-2.11)	1.28 (0.82-1.97)
	P-trend			*0.191*	*0.177*			*<0.001*	*0.076*			*0.140*	*0.301*
**GLUT1**													
	Low	723	272 (37.6)	1.00 (ref)	1.00 (ref)	87	27 (31.0)	1.00 (ref)	1.00 (ref)	135	47 (34.8)	1.00 (ref)	1.00 (ref)
	Moderate	580	244 (42.1)	1.17 (0.98-1.39)	1.09 (0.92-1.31)	80	31 (38.8)	1.24 (0.74-2.08)	1.04 (0.58-1.87)	118	57 (48.3)	1.56 (1.06-2.29)	1.37 (0.91-2.05)
	High	398	198 (49.7)	1.45 (1.21-1.75)	1.20 (0.99-1.45)	55	24 (43.6)	1.54 (0.89-2.67)	0.78 (0.40-1.51)	75	33 (44.0)	1.45 (0.93-2.27)	1.06 (0.64-1.74)
	P-trend			*<0.001*	*0.059*			*0.124*	*0.487*			*0.058*	*0.602*
**LDHA**													
	Low	582	252 (43.3)	1.00 (ref)	1.00 (ref)	84	29 (34.5)	1.00 (ref)	1.00 (ref)	115	50 (43.4)	1.00 (ref)	1.00 (ref)
	Moderate	789	340 (43.1)	1.00 (0.85-1.18)	1.04 (0.88-1.24)	113	39 (34.5)	0.98 (0.60-1.58)	0.87 (0.52-1.45)	173	73 (42.2)	0.99 (0.69-1.42)	0.96 (0.64-1.42)
	High	330	122 (37.0)	0.83 (0.67-1.04)	0.99 (0.78-1.26)	25	14 (56.0)	1.62 (0.86-3.07)	1.02 (0.45-2.35)	40	14 (35.0)	0.84 (0.46-1.52)	0.67 (0.35-1.30)
	P-trend			*0.154*	*0.950*			*0.262*	*0.851*			*0.638*	*0.317*
**MCT4**													
	Low	834	386 (46.3)	1.00 (ref)	1.00 (ref)	126	46 (36.5)	1.00 (ref)	1.00 (ref)	185	77 (41.6)	1.00 (ref)	1.00 (ref)
	Moderate	466	179 (38.4)	0.80 (0.67-0.96)	0.91 (0.76-1.09)	65	23 (35.4)	1.01 (0.61-1.66)	1.12 (0.62-2.02)	102	39 (38.2)	0.83 (0.56-1.22)	1.17 (0.73-1.86)
	High	401	149 (37.2)	0.78 (0.64-0.94)	0.98 (0.79-1.21)	31	13 (41.9)	1.18 (0.64-2.18)	1.35 (0.67-2.73)	41	21 (51.2)	1.15 (0.71-1.86)	1.53 (0.88-2.66)
	P-trend			*0.003*	*0.675*			*0.661*	*0.405*			*0.950*	*0.144*
**PKM2**													
	Low	760	302 (39.7)	1.00 (ref)	1.00 (ref)	118	40 (33.9)	1.00 (ref)	1.00 (ref)	171	68 (39.8)	1.00 (ref)	1.00 (ref)
	Moderate	455	200 (44.0)	1.15 (0.96-1.38)	1.07 (0.89-1.28)	57	24 (42.1)	1.34 (0.81-2.23)	1.49 (0.82-2.69)	86	39 (45.3)	1.23 (0.83-1.82)	1.32 (0.87-2.00)
	High	486	212 (43.6)	1.13 (0.95-1.35)	1.05 (0.87-1.27)	47	18 (38.3)	1.25 (0.72-2.18)	1.50 (0.80-2.82)	71	30 (42.3)	1.19 (0.77-1.83)	1.32 (0.82-2.15)
	P-trend			*0.132*	*0.545*			*0.326*	*0.138*			*0.335*	*0.179*
**p53**													
	Low	863	351 (40.7)	1.00 (ref)	1.00 (ref)	109	37 (33.9)	1.00 (ref)	1.00 (ref)	142	58 (40.8)	1.00 (ref)	1.00 (ref)
	Moderate	209	92 (44.0)	1.10 (0.87-1.38)	1.18 (0.93-1.49)	13	5 (38.5)	1.20 (0.47-3.06)	0.61 (0.20-1.88)	30	15 (50.0)	1.19 (0.68-2.11)	1.47 (0.80-2.72)
	High	629	271 (43.1)	1.07 (0.91-1.25)	0.95 (0.81-1.12)	100	40 (40.0)	1.33 (0.85-2.08)	0.96 (0.58-1.59)	156	64 (41.0)	1.04 (0.73-1.49)	0.95 (0.65-1.39)
	P-trend			*0.407*	*0.619*			*0.209*	*0.868*			*0.815*	*0.756*
**PTEN**													
	Low	159	66 (41.5)	1.26 (0.96-1.66)	1.14 (0.86-1.51)	12	7 (58.3)	2.40 (1.04-5.54)	1.97 (0.70-5.57)	16	8 (50.0)	1.30 (0.92-1.85)	1.44 (0.66-3.18)
	Moderate	877	406 (46.3)	1.36 (1.16-1.60)	1.12 (0.94-1.33)	123	49 (39.8)	1.42 (0.88-2.28)	1.40 (0.79-2.48)	171	76 (44.4)	1.30 (0.92-1.85)	1.07 (0.72-1.61)
	High	665	242 (36.4)	1.00 (ref)	1.00 (ref)	87	26 (29.9)	1.00 (ref)	1.00 (ref)	141	53 (37.6)	1.00 (ref)	1.00 (ref)
	P-trend			*0.002*	*0.195*			*0.036*	*0.148*			*0.103*	*0.451*

[1]Adjusted for *a priori* defined confounders, including age at diagnosis (years), sex (male, female), tumour location (colon, rectum, rectosigmoid), pTNM stage (I, II, III, IV, unknown), differentiation grade (well, moderate, poor/undifferentiated, unknown), MMR deficiency (no, yes, unknown), adjuvant therapy (no, yes, unknown), and additionally adjusted for the expression levels of all individual proteins (LDHA: low, moderate, high; GLUT1: low, moderate, high; PKM2: low, moderate, high; MCT4: low, moderate high; PTEN: low, moderate, high; p53: low, moderate, high).

[2]Adjusted for *a priori* defined confounders, including age at diagnosis (years), sex (male, female), tumour location (colon, rectum, rectosigmoid), pTNM stage (I, II, III, IV, unknown), differentiation grade (well, moderate, poor/undifferentiated, unknown), MMR deficiency (no, yes, unknown), adjuvant therapy (no, yes, unknown)

Table S3. Univariable and multivariable-adjusted hazard ratios (HRs) and 95% confidence intervals (CIs) for associations between the expression levels (low, moderate, and high) of individual Warburg-related proteins (LDHA, GLUT1, MCT4, PKM2, p53, and PTEN) and overall survival, according to tumour location (colon, rectosigmoid, and rectum).

	**Colon**				**Rectosigmoid**				**Rectum**			
			**HR (95% CI)**				**HR (95% CI)**				**HR (95% CI)**	
	**N**	**Deaths (%)**	**Univariable**	**Multivariable-adjusted[1]**	**N**	**Deaths (%)**	**Univariable**	**Multivariable-adjusted[1]**	**N**	**Deaths (%)**	**Univariable**	**Multivariable-adjusted[1]**
**Warburg subtypes**												
Low	467	299 (64.0)	1.00 (ref)	1.00 (ref)[2]	77	47 (61.0)	1.00 (ref)	1.00 (ref)[2]	108	56 (51.9)	1.00 (ref)	1.00 (ref)[2]
Moderate	606	397 (65.5)	1.05 (0.91-1.23)	1.01 (0.89-1.17)	77	48 (62.3)	1.05 (0.70-1.56)	0.99 (0.65-1.50)	119	69 (58.0)	1.11 (0.78-1.58)	0.96 (0.67-1.38)
High	628	424 (67.5)	1.11 (0.96-1.29)	1.12 (0.96-1.30)	68	52 (76.5)	1.84 (1.24-2.74)	1.47 (0.96-2.26)	101	71 (70.3)	1.64 (1.16-2.34)	1.58 (1.10-2.28)
P-trend			*0.161*	*0.125*			*0.003*	*0.087*			*0.005*	*0.015*
**GLUT1**												
Low	723	457 (63.2)	1.00 (ref)	1.00 (ref)	87	57 (65.5)	1.00 (ref)	1.00 (ref)	135	76 (56.3)	1.00 (ref)	1.00 (ref)
Moderate	580	379 (65.3)	1.09 (0.95-1.24)	1.05 (0.91-1.21)	80	51 (63.8)	0.97 (0.66-1.42)	0.99 (0.65-1.49)	118	73 (61.9)	1.25 (0.91-1.73)	1.12 (0.80-1.58)
High	398	284 (71.4)	1.27 (1.10-1.47)	1.11 (0.95-1.30)	55	39 (70.9)	1.23 (0.82-1.84)	0.85 (0.53-1.35)	75	47 (62.7)	1.29 (0.90-1.86)	0.99 (0.66-1.49)
P-trend			*0.002*	*0.174*			*0.382*	*0.511*			*0.133*	*0.906*
**LDHA**												
Low	582	380 (65.3)	1.00 (ref)	1.00 (ref)	84	53 (63.1)	1.00 (ref)	1.00 (ref)	115	67 (58.3)	1.00 (ref)	1.00 (ref)
Moderate	789	524 (66.4)	1.03 (0.90-1.18)	1.05 (0.91-1.20)	113	77 (68.1)	1.08 (0.76-1.53)	1.01 (0.69-1.47)	173	105 (60.7)	1.07 (0.79-1.46)	1.06 (0.75-1.48)
High	330	216 (65.5)	0.98 (0.83-1.16)	1.08 (0.90-1.30)	25	17 (68.0)	1.10 (0.63-1.89)	0.92 (0.48-1.76)	40	24 (60.0)	1.09 (0.68-1.73)	0.99 (0.59-1.67)
P-trend			*0.939*	*0.386*			*0.662*	*0.884*			*0.653*	*0.917*
**MCT4**												
Low	834	573 (68.7)	1.00 (ref)	1.00 (ref)	126	81 (64.3)	1.00 (ref)	1.00 (ref)	185	108 (58.4)	1.00 (ref)	1.00 (ref)
Moderate	466	298 (63.9)	0.89 (0.77-1.02)	0.98 (0.85-1.14)	65	46 (70.8)	1.17 (0.81-1.68)	1.31 (0.86-1.98)	102	60 (58.8)	0.92 (0.67-1.26)	1.06 (0.73-1.53)
High	401	249 (62.1)	0.86 (0.74-0.99)	0.99 (0.83-1.17)	31	20 (64.5)	1.04 (0.63-1.69)	1.26 (0.73-2.16)	41	28 (68.3)	1.13 (0.74-1.71)	1.19 (0.75-1.91)
P-trend			*0.027*	*0.872*			*0.658*	*0.254*			*0.826*	*0.479*
**PKM2**												
Low	760	492 (64.7)	1.00 (ref)	1.00 (ref)	118	76 (64.4)	1.00 (ref)	1.00 (ref)	171	98 (57.3)	1.00 (ref)	1.00 (ref)
Moderate	455	300 (65.9)	1.06 (0.92-1.22)	1.01 (0.87-1.17)	57	40 (70.2)	1.20 (0.82-1.77)	1.36 (0.88-2.09)	86	53 (61.6)	1.18 (0.85-1.65)	1.30 (0.91-1.85)
High	486	328 (67.5)	1.08 (0.94-1.24)	1.03 (0.89-1.20)	47	31 (66.0)	1.14 (0.75-1.73)	1.25 (0.78-1.99)	71	45 (63.4)	1.26 (0.88-1.79)	1.41 (0.95-2.09)
P-trend			*0.276*	*0.676*			*0.428*	*0.222*			*0.169*	*0.064*
**p53**												
Low	863	565 (65.4)	1.00 (ref)	1.00 (ref)	109	68 (62.4)	1.00 (ref)	1.00 (ref)	142	78 (54.9)	1.00 (ref)	1.00 (ref)
Moderate	209	136 (65.1)	1.01 (0.84-1.22)	1.07 (0.88-1.29)	13	8 (61.5)	1.06 (0.51-2.20)	0.76 (0.34-1.68)	30	17 (56.7)	1.01 (0.60-1.70)	1.14 (0.65-2.00)
High	629	419 (66.6)	1.03 (0.90-1.16)	0.95 (0.83-1.08)	100	71 (71.0)	1.31 (0.94-1.83)	1.06 (0.74-1.52)	156	101 (64.7)	1.23 (0.92-1.66)	1.16 (0.85-1.60)
P-trend			*0.683*	*0.460*			*0.113*	*0.768*			*0.161*	*0.351*
**PTEN**												
Low	159	101 (63.5)	1.14 (0.92-1.42)	1.14 (0.91-1.42)	12	10 (83.3)	1.83 (0.93-3.59)	2.07 (0.96-4.48)	16	12 (75.0)	1.52 (0.83-2.78)	1.27 (0.65-2.47)
Moderate	877	610 (69.6)	1.23 (1.09-1.39)	1.13 (0.99-1.29)	123	84 (68.3)	1.23 (0.87-1.73)	1.47 (0.98-2.21)	171	103 (60.2)	1.15 (0.86-1.54)	1.04 (0.75-1.44)
High	665	409 (61.5)	1.00 (ref)	1.00 (ref)	87	53 (60.9)	1.00 (ref)	1.00 (ref)	141	81 (57.4)	1.00 (ref)	1.00 (ref)
P-trend			*0.012*	*0.088*			*0.080*	*0.027*			*0.157*	*0.589*

[1]Adjusted for *a priori* defined confounders, including age at diagnosis (years), pTNM stage (I, II, III, IV, unknown), differentiation grade (well, moderate, poor/undifferentiated, unknown), MMR deficiency (no, yes, unknown), adjuvant therapy (no, yes, unknown), and additionally adjusted for the expression levels of all individual proteins (LDHA: low, moderate, high; GLUT1: low, moderate, high; PKM2: low, moderate, high; MCT4: low, moderate high; PTEN: low, moderate, high; p53: low, moderate, high).
[2]Adjusted for *a priori* defined confounders, including age at diagnosis (years), sex (male, female), pTNM stage (I, II, III, IV, unknown), differentiation grade (well, moderate, poor/undifferentiated, unknown), MMR deficiency (no, yes, unknown), adjuvant therapy (no, yes, unknown)

Table S4. Univariable and multivariable-adjusted hazard ratios (HRs) and 95% confidence intervals (CIs) for associations between the expression levels (low, moderate, and high) of individual Warburg-related proteins (LDHA, GLUT1, MCT4, PKM2, p53, and PTEN) and CRC-specific survival, according to disease stage (pTNM stage I–IV).

	pTNM stage I				pTNM stage II				pTNM stage III				pTNM stage IV			
	N	CRC deaths (%)	HR (95% CI) Univariable	HR (95% CI) Multivariable-adjusted[1]	N	CRC deaths (%)	HR (95% CI) Univariable	HR (95% CI) Multivariable-adjusted[1]	N	CRC deaths (%)	HR (95% CI) Univariable	HR (95% CI) Multivariable-adjusted[1]	N	CRC deaths (%)	HR (95% CI) Univariable	HR (95% CI) Multivariable-adjusted[1]
**Warburg subtypes**																
Low	155	24 (15.5)	1.00 (ref)	1.00 (ref)[2]	247	64 (25.9)	1.00 (ref)	1.00 (ref)[2]	152	76 (50.0)	1.00 (ref)	1.00 (ref)[2]	80	72 (90.0)	1.00 (ref)	1.00 (ref)[2]
Moderate	151	25 (16.6)	1.06 (0.61-1.86)	1.07 (0.61-1.88)	297	86 (29.0)	1.14 (0.83-1.58)	1.13 (0.81-1.56)	217	107 (49.3)	0.96 (0.71-1.29)	0.99 (0.73-1.33)	118	109 (92.4)	1.07 (0.79-1.44)	1.00 (0.74-1.35)
High	118	18 (15.3)	1.01 (0.55-1.86)	1.09 (0.58-2.05)	324	87 (26.9)	1.04 (0.76-1.44)	1.04 (0.75-1.44)	213	123 (57.7)	1.39 (1.04-1.84)	1.42 (1.06-1.90)	127	115 (90.6)	1.33 (0.99-1.78)	1.11 (0.81-1.51)
P-trend			*0.955*	*0.780*			*0.851*	*0.872*			*0.015*	*0.010*			*0.052*	*0.507*
**GLUT1**																
Low	213	31 (14.6)	1.00 (ref)	1.00 (ref)	360	89 (24.7)	1.00 (ref)	1.00 (ref)	228	108 (47.4)	1.00 (ref)	1.00 (ref)	119	107 (89.9)	1.00 (ref)	1.00 (ref)
Moderate	147	29 (19.7)	1.36 (0.82-2.26)	1.24 (0.73-2.09)	312	85 (27.2)	1.16 (0.86-1.56)	1.11 (0.82-1.50)	189	102 (54.0)	1.14 (0.87-1.49)	1.09 (0.82-1.44)	111	104 (93.7)	1.24 (0.94-1.62)	0.93 (0.69-1.25)
High	64	7 (10.9)	0.69 (0.30-1.57)	0.71 (0.31-1.63)	197	63 (32.1)	1.35 (0.98-1.87)	1.23 (0.88-1.72)	165	96 (58.2)	1.43 (1.08-1.88)	1.31 (0.98-1.75)	95	85 (89.5)	1.10 (0.83-1.47)	0.84 (0.61-1.16)
P-trend			*0.789*	*0.727*			*0.066*	*0.220*			*0.013*	*0.076*			*0.436*	*0.296*
**LDHA**																
Low	146	23 (15.8)	1.00 (ref)	1.00 (ref)	303	92 (30.4)	1.00 (ref)	1.00 (ref)	208	111 (53.4)	1.00 (ref)	1.00 (ref)	103	95 (92.2)	1.00 (ref)	1.00 (ref)
Moderate	217	34 (15.7)	0.98 (0.58-1.67)	0.96 (0.56-1.65)	403	116 (28.8)	0.96 (0.73-1.27)	1.03 (0.77-1.38)	269	142 (52.8)	0.98 (0.76-1.25)	0.97 (0.75-1.27)	161	146 (90.7)	0.99 (0.76-1.28)	1.06 (0.80-1.41)
High	61	10 (16.4)	1.02 (0.48-2.13)	1.07 (0.48-2.36)	162	29 (17.9)	0.56 (0.37-0.85)	0.65 (0.41-1.03)	105	53 (50.5)	0.96 (0.69-1.33)	1.00 (0.70-1.44)	61	55 (90.2)	0.96 (0.69-1.33)	1.19 (0.82-1.73)
P-trend			*0.991*	*0.947*			*0.017*	*0.163*			*0.786*	*0.959*			*0.802*	*0.378*
**MCT4**																
Low	209	33 (15.8)	1.00 (ref)	1.00 (ref)	421	123 (29.2)	1.00 (ref)	1.00 (ref)	307	170 (55.4)	1.00 (ref)	1.00 (ref)	181	169 (93.4)	1.00 (ref)	1.00 (ref)
Moderate	128	21 (16.4)	0.99 (0.57-1.72)	1.05 (0.59-1.84)	243	60 (24.7)	0.83 (0.61-1.12)	0.84 (0.61-1.16)	171	86 (50.3)	0.93 (0.72-1.20)	0.90 (0.68-1.18)	77	67 (87.0)	1.04 (0.79-1.39)	1.03 (0.75-1.41)
High	87	13 (14.9)	0.92 (0.48-1.75)	1.27 (0.62-2.62)	204	54 (26.5)	0.88 (0.64-1.21)	1.07 (0.75-1.53)	104	50 (48.1)	0.92 (0.67-1.26)	0.98 (0.68-1.41)	67	60 (89.6)	1.09 (0.81-1.47)	0.95 (0.66-1.36)
P-trend			*0.815*	*0.552*			*0.320*	*0.933*			*0.538*	*0.723*			*0.543*	*0.832*
**PKM2**																
Low	210	34 (16.2)	1.00 (ref)	1.00 (ref)	394	100 (25.3)	1.00 (ref)	1.00 (ref)	274	136 (49.6)	1.00 (ref)	1.00 (ref)	134	121 (90.3)	1.00 (ref)	1.00 (ref)
Moderate	119	23 (19.3)	1.23 (0.73-2.10)	1.30 (0.74-2.27)	227	61 (26.9)	1.07 (0.77-1.46)	1.09 (0.79-1.51)	151	86 (57.0)	1.29 (0.98-1.69)	1.23 (0.93-1.63)	96	89 (92.7)	1.00 (0.76-1.32)	1.02 (0.76-1.36)
High	95	10 (10.5)	0.67 (0.33-1.37)	0.73 (0.34-1.54)	245	76 (31.0)	1.25 (0.92-1.68)	1.27 (0.93-1.75)	157	84 (52.5)	1.17 (0.89-1.53)	1.20 (0.89-1.61)	95	86 (90.5)	1.16 (0.88-1.54)	1.05 (0.77-1.44)
P-trend			*0.451*	*0.638*			*0.157*	*0.139*			*0.188*	*0.180*			*0.313*	*0.753*
**p53**																
Low	221	38 (17.2)	1.00 (ref)	1.00 (ref)	442	126 (28.5)	1.00 (ref)	1.00 (ref)	279	139 (49.8)	1.00 (ref)	1.00 (ref)	152	133 (87.5)	1.00 (ref)	1.00 (ref)
Moderate	36	3 (8.3)	0.47 (0.14-1.51)	0.57 (0.17-1.92)	106	30 (28.3)	0.98 (0.66-1.46)	0.99 (0.66-1.48)	60	33 (55.0)	1.06 (0.72-1.55)	1.12 (0.76-1.65)	42	41 (97.6)	1.67 (1.17-2.39)	1.77 (1.17-2.66)
High	167	26 (15.6)	0.91 (0.55-1.50)	0.78 (0.47-1.32)	320	81 (25.3)	0.88 (0.67-1.17)	0.79 (0.59-1.05)	243	134 (55.1)	1.14 (0.90-1.44)	1.13 (0.88-1.44)	131	122 (93.1)	1.12 (0.87-1.43)	1.13 (0.86-1.47)
P-trend			*0.663*	*0.338*			*0.386*	*0.116*			*0.289*	*0.337*			*0.373*	*0.441*
**PTEN**																
Low	23	5 (21.7)	1.62 (0.63-4.17)	2.18 (0.80-5.96)	78	19 (24.4)	1.08 (0.65-1.79)	0.95 (0.56-1.59)	49	23 (46.9)	1.14 (0.73-1.79)	1.16 (0.72-1.85)	32	31 (96.9)	1.53 (1.02-2.31)	0.97 (0.62-1.52)
Moderate	191	30 (15.7)	1.00 (0.61-1.64)	1.15 (0.67-1.95)	444	141 (31.8)	1.51 (1.15-2.00)	1.35 (1.00-1.82)	320	177 (55.3)	1.24 (0.97-1.58)	1.24 (0.96-1.61)	185	169 (91.4)	1.00 (0.78-1.29)	0.88 (0.66-1.16)
High	210	32 (15.2)	1.00 (ref)	1.00 (ref)	346	77 (22.3)	1.00 (ref)	1.00 (ref)	213	106 (49.8)	1.00 (ref)	1.00 (ref)	108	96 (88.9)	1.00 (ref)	1.00 (ref)
P-trend			*0.565*	*0.232*			*0.082*	*0.425*			*0.170*	*0.185*			*0.172*	*0.587*

[1]Adjusted for *a priori* defined confounders, including age at diagnosis (years), sex (male, female), tumour location (colon, rectum, rectosigmoid), pTNM stage (I, II, III, IV, unknown), differentiation grade (well, moderate, poor/undifferentiated, unknown), MMR deficiency (no, yes, unknown), adjuvant therapy (no, yes, unknown), and additionally adjusted for the expression levels of all individual proteins (LDHA: low, moderate, high; GLUT1: low, moderate, high; PKM2: low, moderate, high; MCT4: low, moderate high; PTEN: low, moderate, high; p53: low, moderate, high).

[2]Adjusted for *a priori* defined confounders, including age at diagnosis (years), sex (male, female), tumour location (colon, rectum, rectosigmoid), pTNM stage (I, II, III, IV, unknown), differentiation grade (well, moderate, poor/undifferentiated, unknown), MMR deficiency (no, yes, unknown), adjuvant therapy (no, yes, unknown)

Table S5. Univariable and multivariable-adjusted hazard ratios (HRs) and 95% confidence intervals (CIs) for associations between the expression levels (low, moderate, and high) of individual Warburg-related proteins (LDHA, GLUT1, MCT4, PKM2, p53, and PTEN) and overall survival, according to disease stage (pTNM stage I–IV).

	pTNM stage I				pTNM stage II				pTNM stage III				pTNM stage IV			
	N	Deaths (%)	HR (95% CI)		N	Deaths (%)	HR (95% CI)		N	Deaths (%)	HR (95% CI)		N	Deaths (%)	HR (95% CI)	
			Univariable	Multivariable-adjusted[1]			Univariable	Multivariable-adjusted[1]			Univariable	Multivariable-adjusted[1]			Univariable	Multivariable-adjusted[1]
**Warburg subtypes**																
Low	155	74 (47.7)	1.00 (ref)	1.00 (ref)[2]	247	134 (54.3)	1.00 (ref)	1.00 (ref)[2]	152	103 (67.8)	1.00 (ref)	1.00 (ref)[2]	80	79 (98.8)	1.00 (ref)	1.00 (ref)[2]
Moderate	151	63 (41.7)	0.87 (0.62-1.21)	0.86 (0.61-1.20)	297	165 (55.6)	1.05 (0.84-1.32)	1.00 (0.80-1.26)	217	155 (71.4)	1.02 (0.80-1.31)	1.08 (0.84-1.39)	118	117 (99.2)	1.03 (0.77-1.37)	0.95 (0.71-1.27)
High	118	57 (48.3)	1.04 (0.74-1.47)	0.95 (0.66-1.37)	324	186 (57.4)	1.08 (0.86-1.35)	1.07 (0.85-1.34)	213	167 (78.4)	1.40 (1.10-1.79)	1.45 (1.13-1.86)	127	127 (100.0)	1.35 (1.02-1.79)	1.12 (0.83-1.52)
P-trend			*0.895*	*0.709*			*0.508*	*0.538*			*0.004*	*0.002*			*0.029*	*0.400*
**GLUT1**																
Low	213	98 (46.0)	1.00 (ref)	1.00 (ref)	360	194 (53.9)	1.00 (ref)	1.00 (ref)	228	162 (71.1)	1.00 (ref)	1.00 (ref)	119	118 (99.2)	1.00 (ref)	1.00 (ref)
Moderate	147	69 (46.9)	1.02 (0.75-1.39)	0.97 (0.70-1.33)	312	177 (56.7)	1.12 (0.91-1.37)	1.10 (0.90-1.36)	189	134 (70.9)	1.01 (0.80-1.26)	0.95 (0.75-1.21)	111	111 (100.0)	1.21 (0.93-1.57)	0.93 (0.70-1.25)
High	64	27 (42.2)	0.84 (0.55-1.28)	0.85 (0.55-1.32)	197	114 (58.2)	1.13 (0.90-1.43)	1.08 (0.85-1.37)	165	129 (78.2)	1.30 (1.03-1.64)	1.19 (0.93-1.52)	95	94 (98.9)	1.10 (0.84-1.45)	0.87 (0.64-1.18)
P-trend			*0.531*	*0.498*			*0.238*	*0.449*			*0.036*	*0.204*			*0.428*	*0.366*
**LDHA**																
Low	146	67 (45.9)	1.00 (ref)	1.00 (ref)	303	173 (57.1)	1.00 (ref)	1.00 (ref)	208	144 (69.2)	1.00 (ref)	1.00 (ref)	103	103 (100.0)	1.00 (ref)	1.00 (ref)
Moderate	217	99 (45.6)	0.99 (0.72-1.35)	0.97 (0.70-1.33)	403	227 (56.3)	1.01 (0.83-1.23)	1.00 (0.81-1.23)	269	202 (75.1)	1.09 (0.88-1.34)	1.08 (0.86-1.36)	161	159 (98.8)	0.99 (0.77-1.27)	1.05 (0.80-1.38)
High	61	28 (45.9)	0.99 (0.64-1.54)	1.01 (0.62-1.65)	162	85 (52.4)	0.87 (0.67-1.13)	0.92 (0.69-1.23)	105	79 (75.2)	1.11 (0.84-1.46)	1.17 (0.86-1.59)	61	61 (100.0)	0.97 (0.71-1.34)	1.19 (0.83-1.70)
P-trend			*0.955*	*0.967*			*0.356*	*0.644*			*0.407*	*0.309*			*0.874*	*0.365*
**MCT4**																
Low	209	102 (48.8)	1.00 (ref)	1.00 (ref)	421	241 (57.2)	1.00 (ref)	1.00 (ref)	307	221 (72.0)	1.00 (ref)	1.00 (ref)	181	180 (99.4)	1.00 (ref)	1.00 (ref)
Moderate	128	56 (43.8)	0.85 (0.62-1.18)	0.83 (0.59-1.16)	243	134 (55.1)	0.94 (0.76-1.16)	0.97 (0.78-1.21)	171	129 (75.4)	1.08 (0.87-1.34)	1.04 (0.83-1.32)	77	76 (98.7)	1.11 (0.85-1.45)	1.12 (0.83-1.51)
High	87	36 (41.4)	0.81 (0.56-1.19)	0.79 (0.51-1.22)	204	110 (53.9)	0.91 (0.73-1.14)	1.03 (0.80-1.33)	104	75 (72.1)	1.06 (0.82-1.38)	1.12 (0.82-1.52)	67	67 (100.0)	1.14 (0.86-1.51)	0.99 (0.70-1.39)
P-trend			*0.230*	*0.204*			*0.387*	*0.888*			*0.547*	*0.475*			*0.321*	*0.926*
**PKM2**																
Low	210	97 (46.2)	1.00 (ref)	1.00 (ref)	394	221 (55.8)	1.00 (ref)	1.00 (ref)	274	192 (70.1)	1.00 (ref)	1.00 (ref)	134	133 (99.3)	1.00 (ref)	1.00 (ref)
Moderate	119	52 (43.7)	0.98 (0.70-1.37)	1.01 (0.71-1.42)	227	122 (53.7)	0.97 (0.78-1.21)	0.98 (0.78-1.22)	151	119 (78.8)	1.27 (1.01-1.59)	1.21 (0.95-1.53)	96	95 (99.0)	0.97 (0.75-1.27)	0.98 (0.74-1.30)
High	95	45 (47.4)	1.05 (0.74-1.50)	1.19 (0.82-1.72)	245	142 (58.0)	1.07 (0.86-1.32)	1.08 (0.87-1.35)	157	114 (72.6)	1.12 (0.89-1.42)	1.13 (0.88-1.45)	95	95 (100.0)	1.18 (0.91-1.54)	1.04 (0.77-1.41)
P-trend			*0.832*	*0.413*			*0.600*	*0.531*			*0.219*	*0.274*			*0.261*	*0.823*
**p53**																
Low	221	104 (47.1)	1.00 (ref)	1.00 (ref)	442	240 (54.3)	1.00 (ref)	1.00 (ref)	279	202 (72.4)	1.00 (ref)	1.00 (ref)	152	150 (98.7)	1.00 (ref)	1.00 (ref)
Moderate	36	12 (33.3)	0.66 (0.36-1.20)	0.69 (0.37-1.29)	106	57 (53.8)	0.99 (0.74-1.32)	0.94 (0.70-1.27)	60	43 (71.7)	0.95 (0.68-1.32)	0.99 (0.71-1.39)	42	42 (100.0)	1.53 (1.08-2.17)	1.58 (1.06-2.35)
High	167	78 (46.7)	1.00 (0.74-1.34)	0.93 (0.68-1.27)	320	188 (58.8)	1.08 (0.89-1.31)	0.98 (0.80-1.20)	243	180 (74.1)	1.05 (0.86-1.28)	1.02 (0.83-1.26)	131	131 (100.0)	1.07 (0.85-1.36)	1.08 (0.83-1.39)
P-trend			*0.932*	*0.586*			*0.423*	*0.856*			*0.661*	*0.829*			*0.546*	*0.629*
**PTEN**																
Low	23	14 (60.9)	1.56 (0.89-2.73)	1.78 (0.99-1.31)	78	36 (46.2)	0.85 (0.60-1.22)	0.86 (0.60-1.25)	49	37 (75.5)	1.32 (0.92-1.89)	1.46 (1.00-2.12)	32	32 (100.0)	1.45 (0.97-2.16)	0.94 (0.61-1.45)
Moderate	191	82 (42.9)	0.87 (0.65-1.17)	0.96 (0.70-1.31)	444	267 (60.1)	1.23 (1.02-1.48)	1.26 (1.03-1.54)	320	242 (75.6)	1.24 (1.01-1.52)	1.33 (1.06-1.66)	185	184 (99.5)	1.00 (0.78-1.27)	0.90 (0.69-1.18)
High	210	98 (46.7)	1.00 (ref)	1.00 (ref)	346	182 (52.6)	1.00 (ref)	1.00 (ref)	213	146 (68.5)	1.00 (ref)	1.00 (ref)	108	107 (99.1)	1.00 (ref)	1.00 (ref)
P-trend			*0.809*	*0.344*			*0.602*	*0.563*			*0.033*	*0.008*			*0.234*	*0.566*

[1]Adjusted for *a priori* defined confounders, including age at diagnosis (years), sex (male, female), tumour location (colon, rectum, rectosigmoid), pTNM stage (I, II, III, IV, unknown), differentiation grade (well, moderate, poor/undifferentiated, unknown), MMR deficiency (no, yes, unknown), adjuvant therapy (no, yes, unknown), and additionally adjusted for the expression levels of all individual proteins (LDHA: low, moderate, high; GLUT1: low, moderate, high; PKM2: low, moderate, high; MCT4: low, moderate high; PTEN: low, moderate, high; p53: low, moderate, high).
[2]Adjusted for *a priori* defined confounders, including age at diagnosis (years), sex (male, female), tumour location (colon, rectum, rectosigmoid), pTNM stage (I, II, III, IV, unknown), differentiation grade (well, moderate, poor/undifferentiated, unknown), MMR deficiency (no, yes, unknown), adjuvant therapy (no, yes, unknown)
